# Neoadjuvant ipilimumab plus nivolumab therapy as a potential organ preservation strategy in mucosal melanoma: case report

**DOI:** 10.3389/fonc.2024.1301424

**Published:** 2024-03-14

**Authors:** Claire V. Ong, Wolfram Samlowski

**Affiliations:** ^1^Kirk Kerkorian School of Medicine, University of Nevada Las Vegas (UNLV), Las Vegas, NV, United States; ^2^Comprehensive Cancer Centers of Nevada, Las Vegas, NV, United States; ^3^Department of Internal Medicine, University of Nevada School of Medicine, Reno, NV, United States

**Keywords:** neoadjuvant therapy, checkpoint inhibitors, sinonasal melanoma, rectal melanoma, organ preservation

## Abstract

Mucosal melanoma represents an uncommon melanoma subtype. Wide excision has long represented the standard therapeutic approach. Unfortunately, there is a high relapse rate and mortality. Neoadjuvant therapy with ipilimumab plus nivolumab has shown significant activity in cutaneous melanoma. We present two cases of mucosal melanoma, each with potential regional dissemination, who were treated with neoadjuvant immunotherapy with minimal toxicity. Both patients were closely monitored and achieved radiologic and pathologic complete responses. These patients were able to avoid radical surgery and related functional consequences. Both patients remain recurrence-free with protracted follow-up. The potential usefulness of neoadjuvant immunotherapy as an organ preservation strategy in mucosal melanoma deserves further evaluation in prospective clinical trials.

## Introduction

Mucosal melanoma (MM) is an uncommon melanoma subtype, associated with an inferior clinical outcome, compared to cutaneous melanoma (CM) (1). Whenever technically feasible, surgical resection has long been the standard therapy for MM. Complete surgical resection of MM with negative pathological margins has been associated with a somewhat better prognosis (2, 3), but there is no evidence that more radical resection improves overall survival (4). Based on a retrospective study, the 5-year survival rate of MM, including all stages of disease, is only 10-20% when compared to 93% for CM (5–7). This is largely because of delayed detection and a high risk of lymph node or distant metastases due to the lack of anatomic barriers to dissemination (8). An increased risk for local recurrence following resection also represents an additional management challenge, perhaps due to potential multifocal origin of MM (9, 10). In addition, definitive surgical resection frequently results in significant functional and cosmetic deficits.

Immunotherapy using immune checkpoint inhibitor (ICI)-directed antibodies has become an increasingly important treatment option for cutaneous melanoma in recent years. Commonly used ICIs include ipilimumab, an antibody against cytotoxic T lymphocyte antigen 4 (CTLA4). Also, nivolumab and pembrolizumab, monoclonal antibodies against programmed death-1 (PD1) have shown clinical activity (11). Combination therapy using CTLA4 and PD1 antibodies together in cutaneous melanoma has produced further increases in progression-free and overall survival, albeit with increased risk of toxicity (12).

Recently, a pre-operative (neoadjuvant) treatment approach has shown significant promise in regionally advanced cutaneous melanoma. In a pair of phase II studies, this approach has yielded a high percentage of patients who achieved a pathologic complete response or near complete response (13, 14). Patients with virtually complete pathologic responses also appeared to benefit with a very high rate of progression-free survival. In a more recent SWOG 1801 randomized clinical trial, resectable stage III or IV cutaneous melanoma patients were randomized to receive either neoadjuvant pembrolizumab followed by adjuvant therapy or post-surgical adjuvant pembrolizumab. In this trial, the 2-year event-free survival in the neoadjuvant-adjuvant group was significantly higher than that of the adjuvant-only group, 72% compared to 49% (15). These results strongly suggest that neoadjuvant therapy improves outcomes in locoregionally advanced cutaneous melanoma.

We employed this approach in two patients with high-risk locoregional mucosal melanoma. These patients both achieved a dramatic response following neoadjuvant ipilimumab and nivolumab therapy without undergoing a planned radical surgical resection, thus suggesting that this treatment approach may also be useful for an “organ preservation” approach.

## Case presentation

### Case 1

**Figure 1:**
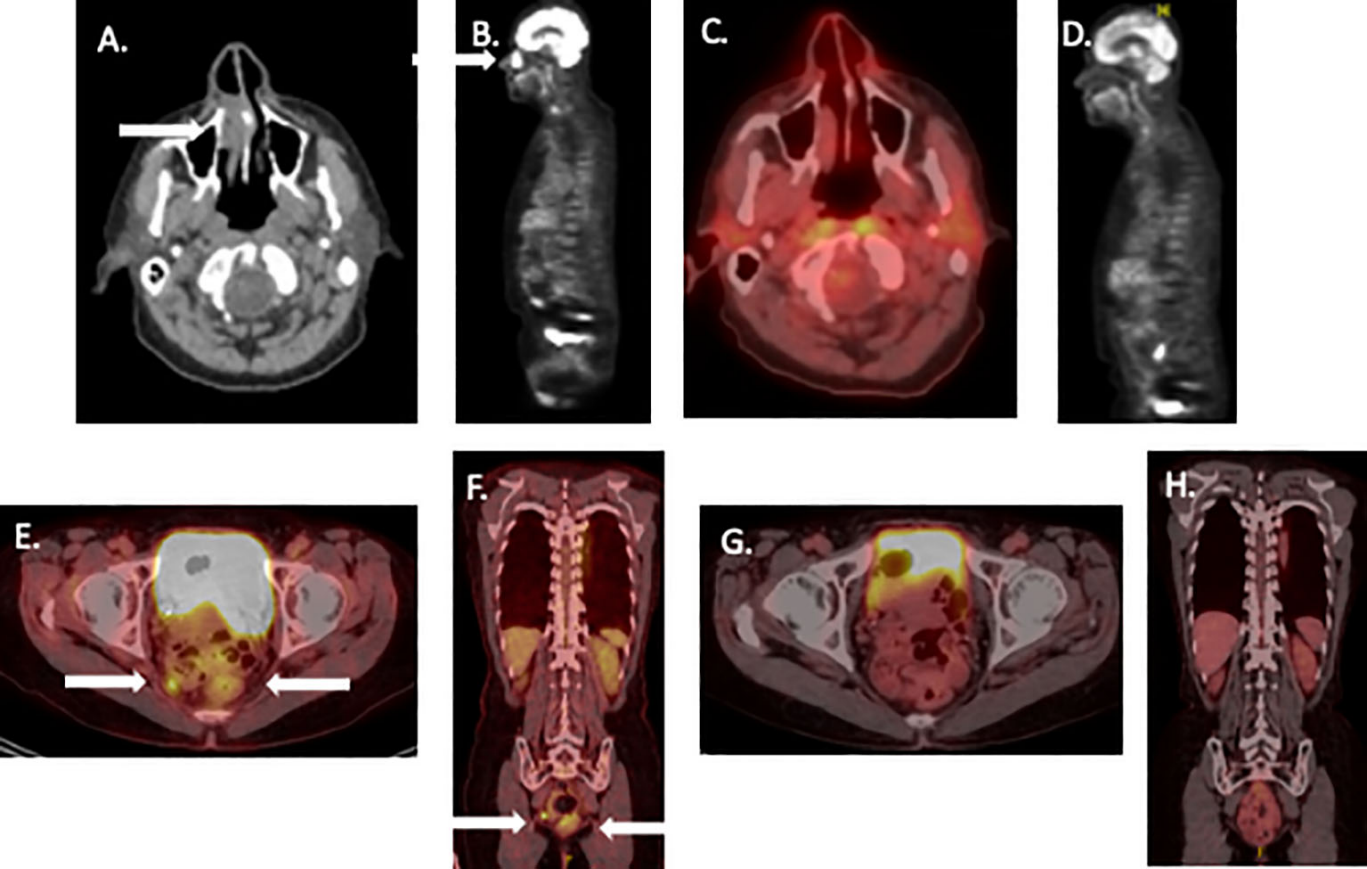
Pre- and post-treatment scans of patients undergoing neoadjuvant therapy for mucosal melanoma. **(A, B)**: Pretreatment PET/CT scan images patient 1 showing nasal melanoma. **(C, D)**: similar PET/CT views 3 years later. **(E, F)**: Pretreatment PET CT of patient 2, **(G, H)** similar PET/CT views showing complete remission 2 years later. White arrows show tumor site on initial images.

A 74-year-old man presented with a locally advanced sinonasal melanoma. The patient had palpated a mass in his right nostril that obstructed his breathing for months. MRI of the head showed a 2.4 x 1.2 x 2.2 cm mass in the right nasal fossa (**Figures 1A, B**). A biopsy showed a poorly differentiated melanoma. This was confirmed by a PET/CT scan. Possible small lung nodules (thought benign) and prominent mediastinal lymph nodes (indeterminate) were identified (stage T3, N0, MX). The patient declined radical surgery for resection of the mass. Biopsies of lung and lymph nodes were not felt feasible. Therefore, a neoadjuvant treatment approach was discussed, with the option of delayed surgery or radiotherapy.

The patient was treated with the alternate regimen of ipilimumab (1 mg/kg) plus nivolumab (3 mg/kg) intravenously every 3 weeks (16). After the 3^rd^ treatment, endoscopic re-evaluation revealed no residual tumor. After the fourth cycle, antitumor response was assessed via PET/CT scan. This suggested a complete resolution of all previous abnormalities. A complete remission was confirmed by biopsy of the previous tumor site (identified by residual abnormal pigmentation).

Patient was continued on nivolumab maintenance (480 mg fixed dose every 4 weeks) for one year. As scans continued to demonstrate a complete remission, treatment was electively discontinued. The patient has remained disease-free for > 42 months (**Figures 1C, D**).

### Case 2

A 62-year-old woman presented with a regionally advanced rectal melanoma. This was diagnosed after the patient complained of a history of hematochezia for many months. Colonoscopy identified a rectal mass. A biopsy revealed a poorly differentiated, SOX10 positive rectal melanoma. A PET/CT confirmed the presence of a 4 cm rectal tumor encroaching on the anus with a 1.2 cm abnormal intrapelvic lymph node (**Figures 1E, F**). Thus, the patient was diagnosed with modified Ballantine stage II, modified AJCC stage III (T3, N1, M0) rectal melanoma (17, 18). A multidisciplinary discussion considered AP-resection; lymph node resection followed by adjuvant therapy versus neoadjuvant immunotherapy for 2 cycles followed by resection. The patient was reluctant to undergo AP resection and colostomy and favored attempted rectal function preservation.

The patient was treated with the same ipilimumab plus nivolumab regimen described above. The patient received 2 cycles of treatment and an abdominal MRI showed significant improvement in the rectal mass and resolution of the pelvic lymph node. Immunotherapy was therefore continued with planned re-evaluation after a 4^th^ dose. Treatment was complicated by a mild rash with extensive pruritus after the 3^rd^ treatment. This was treated with antihistamines and low dose steroids.

Following 4 treatment cycles, tumor was reimaged with PET scan. This appeared to show a complete remission of all disease. The patient underwent limited surgical resection of the area of the prior rectal tumor (identified by residual pigmentation) with sphincter preservation. Pathologic evaluation showed a complete response characterized by abnormal pigment accumulation in macrophages without residual cancer. After the patient recovered from surgery, she received nivolumab maintenance therapy for an additional 6 months. During maintenance therapy, she developed laboratory evidence for hypopituitarism and remained on endocrine replacement therapy. After 2 negative PET/CT scans and a negative endoscopic biopsy of the tumor site, the patient elected to discontinue treatment, based on our local treatment discontinuation protocol (19). The patient continues in an ongoing radiological complete remission for >24 months (**Figures 1G, H**).

## Discussion

In MM, complete surgical resection with negative tumor margins has long been thought to represent the only potential curative treatment. However, complete resection is difficult to achieve due to several challenges. MM often has a multifocal pattern of growth making clear tumor margins harder to delineate. MM also tends to occur in surgically challenging anatomical sites. Furthermore, while complete surgical resection is associated with a somewhat better outcome, there is no evidence that radical resection leads to improved overall survival rates (4). Even if resection with negative tumor margins is achievable, local recurrence within a year post-operation is common and is often accompanied with disseminated disease (20, 21). It should be noted that both of the cases in this report were suspected to have regional lymph node involvment, decreasing the potential for a curative surgical resection. Additionally, patients who undergo radical surgery frequently suffer major debilitating cosmetic and functional deficits.

The theoretical basis for neoadjuvant therapy relies on the likelihood that the preservation of the tumor microenvironment leads to the induction of a more robust anticancer response. Neoadjuvant treatment provides an earlier initiation of systemic therapy to target and eradicate micrometastases, perhaps reducing the potential for metastatic recurrence. The therapeutic effectiveness of an immunotherapy regimen can be assessed in each individual patient, thus providing the ability to tailor subsequent adjuvant therapy based on initial response. In addition, this approach also provides the ability to identify patients with a pathologic CR, which is thought to be a surrogate for improved clinical outcomes (relapse-free and overall survival) without requiring additional surgery. Furthermore, reduction in the extent of a cancer following neoadjuvant treatment may decrease the scope of surgery needed to encompass disease. Theoretical concerns have previously been expressed about the potential for neoadjuvant therapy to delay or prevent planned surgery due to side-effects or tumor progression. In practice, this has proven to be an infrequent issue (13–15).

The concept of organ preservation (use of chemo-, immuno- or radiotherapy to avoid of radical surgery with debilitating function consequences) was initially pioneered in squamous cell carcinoma of the head and neck (SCCHN). Dramatic responses of bulky unresectable SCCHN were originally identified in clinical trials following neoadjuvant concurrent chemo- and radiotherapy in the 1980’s (22). This approach has resulted in a significant percentage of advanced SCCHN patients achieving a complete remission. This led clinical trials of neoadjuvant chemo-radiotherapy as an organ preservation strategy in laryngeal, base of tongue, and hypopharyngeal SCCHN, with avoidance of laryngectomy or glossectomy in a high percentage of patients (23, 24). Due to a significant CR rate and the possibility of surgical salvage, this approach has become a standard current treatment approach in loco-regionally advanced SCCHN (25).

More recently, high clinical response rates and clinical complete remissions were described following pembrolizumab therapy of microsatellite instability-high rectal adenocarcinomas, compared to standard chemotherapy (26). As a high percentage of these patients achieved a pathologically confirmed complete remission, radical anterior-posterior resection of the anal region was avoided in some patients, with preservation of sphincter function (27, 28). This approach is now undergoing further testing as an organ preservation strategy.

Neoadjuvant treatment is demonstrating notable success in advanced CM patients. In the OpACIN and OpACIN-neo studies, stage III melanoma patients were treated with neoadjuvant nivolumab (anti-PD1) plus ipilimumab (anti-CTLA4) using several dosing schedules. The 2-year response free survival for patients that achieved a pathologic response was 97% compared to 36% for those without a major pathologic response, with an overall estimated relapse-free survival (RFS) of 84% (Rozeman et. al., 2021). This approach was further tested in the PRADO trial, in which 99 stage III cutaneous melanoma patients underwent neoadjuvant nivolumab plus ipilimumab therapy and were given tailored treatment options depending on their pathological response rate. Of the study participants, 71 of 99 achieved complete pathological response and 69 of these patients were able to undergo a therapeutic lymph node dissection. In patients with a complete pathologic response, further adjuvant therapy was omitted from their treatment plan. These patients reported significantly higher health-related quality of life outcomes and lower symptom burden. An estimated 24-month relapse-free survival (RFS) of 93% was observed in patients with major pathologic response (Reijiers et. al., 2022). This result was further supported by the recent SWOG 1801 clinical trial. In this randomized phase II clinical trial, 154 patients with potentially resectable stage IIIb to IVc melanoma were randomly assigned to 3 cycles of neoadjuvant pembrolizumab over 9 weeks, followed by surgery with adjuvant pembrolizumab. Alternatively, patients were treated with surgical resection followed by adjuvant pembrolizumab. The treatment duration was equivalent in the two groups. The patient group that received neoadjuvant therapy demonstrated a significantly longer 2-year event-free survival (72%), compared to the adjuvant group (49%). Additionally, only 12% of the patients receiving neoadjuvant and adjuvant ICI therapy experienced a treatment related adverse event (Patel et. al., 2023). Planned surgery was only rarely delayed or omitted in these three trials, due to toxicity or tumor progression. With such significantly improved outcomes in advanced cutaneous melanoma patients, neoadjuvant ICI therapy offers a promising treatment possibility for MM.

We recognize that retrospective studies have suggested that cutaneous melanoma patients demonstrated a longer median progression-free survival and higher objective response rates compared to MM following either ICI monotherapy or combination therapy (29, 30). Thus, we employed a modified ipilimumab plus nivolumab regimen associated with reduced toxicity in our patients (16). Unlike cutaneous melanoma, mucosal melanomas rarely express somatic mutations in BRAF, which limits targeted therapy options (7). Even in the subset of MM with C-KIT mutations, targeted therapy has demonstrated a relatively low response rate, with a relatively short response duration (31–35).

The plan was for patients to receive 2 cycles of therapy prior to restaging scans and endoscopy, followed by potential surgical resection. At interim scans, after 2 cycles of treatment, both patients were found to be responding dramatically. Thus, ipilimumab plus nivolumab induction was continued. Both patients achieved an apparent complete response by subsequent PET/CT after 4 treatment cycles. Endoscopy with biopsies confirmed a pathologic complete response. Both patients underwent a limited resection (based on residual mucosal pigmentation) of the involved site, confirming a complete remission. Following recovery from surgery, both patients continued maintenance PD1 antibody therapy, and treatment was eventually discontinued once confirmatory scans showed an ongoing remission, as previously published (19). It should be noted that we treated 2 other mucosal melanoma patients (vaginal, urethral) with neoadjuvant therapy as they were believed unresectable. Neither responded to treatment. Thus close follow-up of mucosal melanoma patients is imperative. If patients fail to respond after 2 cycles of treatment, prompt surgical salvage should be considered.

In a recent retrospective review of neoadjuvant therapy of mucosal melanoma, Ho et al. identified 36 patients treated with this approach (36). The primary sites of disease were anorectal (53%), urogenital (25%), head and neck (17%), and esophageal (6%). Node positive disease or satellite lesions was present at the time of treatment initiation in 47% of patients. Patients were either treated with PD1 antibodies alone or in combination with ipilimumab. Seventy-eight percent of patients received combined anti-PD1 + anti-CTLA4 therapy. Only 3 patients achieved a complete response and did not undergo planned surgery. Six patients developed disease progression and were unable to have surgery. With a median follow up of 37.9 months, the median event-free survival was 9.2 months with 3-year progression-free survival of 29%. Median overall survival had not been reached, with a 3-year OS rate of 55%. Event-free survival was significantly higher for patients who achieved objective response and for patients with pathologic complete responses. Grade 3 toxicities were reported in 39% of patients. It is not clear why the complete remission rate in this series was unexpectedly low (3%). In part, this may be because 22% of patients in this series were treated with single-agent PD1 or CTLAA4 antibodies rather than more active combination therapy.

In addition, we believe that close monitoring during neoadjuvant therapy is critical. Responses can usually be identified after 2 cycles, thus early restaging with scans and endoscopy is critical to identify patients who should be considered for early surgical salvage. It is doubtful that a six week delay in surgery would have a significant impact on long-term outcome. We suggest that responding patients continue immunotherapy, as they frequently can achieve a complete remission with ongoing therapy (including maintenance PD1therapy). Again, we would stress the need for close radiologic and endoscopic follow-up (including biopsies) to verify complete remissions. Delayed surgical resection of any residual disease detected may also prove useful.

## Conclusions

Ipilimumab plus nivolumab neoadjuvant therapy may be useful in locoregionally advanced MM. Two cycles of iplimumab plus nivolumab treatment over a 6-week period is likely to demonstrate whether significant tumor response is likely and would only minimally delay planned surgery. If patients are found to exhibit a more dramatic response, this therapy can be continued, with the potential to achieve complete remission, without requiring radical surgery. In our opinion, it is important to carefully monitor responses using CT or PET/CT scans. Endoscopic re-evaluation, including biopsies, was also helpful in confirming a pathologic complete response, planning subsequent adjuvant therapy, and eventually allowing planned treatment discontinuation. Thus, neoadjuvant therapy offers a promising treatment option that reduced the need for radical surgical resection, particularly in marginally resectable patients. This approach allowed preservation of normal organ function and avoided radical surgical resection. Further prospective testing of this approach in a multidisciplinary trial is strongly encouraged.

## Data availability statement

The raw data supporting the conclusions of this article will be made available by the authors, without undue reservation.

## Ethics statement

Ethical approval was not required for the study involving humans in accordance with the local legislation and institutional requirements. Written informed consent to participate in this study was not required from the participants or the participants’ legal guardians/next of kin in accordance with the national legislation and the institutional requirements. Written informed consent was obtained from the individual(s) for the publication of any potentially identifiable images or data included in this article.

## Author contributions

CO: Conceptualization, Data curation, Methodology, Writing – original draft, Writing – review & editing. WS: Conceptualization, Formal analysis, Funding acquisition, Investigation, Methodology, Project administration, Supervision, Validation, Writing – review & editing.
